# SERVING UNDERSERVED VETERANS AND THEIR CAREGIVER: DEVELOPING TAILORED CULTURALLY RELEVANT INTERVENTIONS

**DOI:** 10.1093/geroni/igz038.2491

**Published:** 2019-11-08

**Authors:** I Magaly Freytes, Schmitzberger Magda, Rivera-Rivera Naiomi, Lopez Janet, Mylott Daniela, Motta-Valencia Keryl, Constance R Uphold

**Affiliations:** 1 North Florida/South Georgia Veterans Health System, Gainesville, Florida, United States; 2 NF/SG VHS CINDRR, Gainesville, Florida, United States; 3 VA CHS, San Juan, PR, United States

## Abstract

Family members of stroke survivors experience high rates of depression and burden. The majority of stroke survivors return to their homes and need assistance to perform activities of daily living. These demands coupled with the lack of preparedness for their new roles lead to a high risk for developing depression and other negative outcomes among caregivers. Studies indicate that Hispanic caregivers report higher levels of depression compared to others. However, no interventions have focused on this population. Our objective is to develop culturally-relevant interventions to help reduce disparities Hispanic Veterans post-stroke and their caregivers. We tailored our Spanish RESCUE intervention for the Puerto Rican population. The goal of the problem-solving telephone support & educational intervention is to reduce caregiver burden and depressive symptoms by teaching them a creative and optimistic approach to solving caregiving related problems. The intervention was developed to reflect specific characteristics of the target population. To enhance the cultural relevance of the intervention, we used recommendations from Key Stakeholders and guidelines from authoritative sources such as: 1) involving persons from the target population in all phases of the project; 2) emphasizing themes valued by the PR culture; 3) assuring that the language and wording of the materials is at appropriate reading level; 4) using certified translators and Spanish-speaking experts, and 5) having Hispanic research members, fluent in Spanish, and knowledgeable about the PR culture conduct the intervention and assessments. The intervention is currently been tested in a RCT.

